# Silicon modifies C:N:P stoichiometry, and increases nutrient use efficiency and productivity of quinoa

**DOI:** 10.1038/s41598-021-89416-9

**Published:** 2021-05-10

**Authors:** Luis Felipe Lata-Tenesaca, Renato de Mello Prado, Marisa de Cássia Piccolo, Dalila Lopes da Silva, José Lucas Farias da Silva

**Affiliations:** 1 School of Agricultural and Veterinarian Sciences, São Paulo State University (UNESP), Jaboticabal, São Paulo 14884-900 Brazil; 2 Center of Nuclear Energy in Agriculture, University of São Paulo, Piracicaba, São Paulo 13400-970 Brazil

**Keywords:** Plant ecology, Plant physiology

## Abstract

Recognizably, silicon has a beneficial effect on plant growth and productivity. In this respect, it is also known that the C, N and, P stoichiometric ratios and nutrient conversion efficiency allow identifying the interactions between elements while helping to understand the role Si plays in plant growth. This study aims to investigate whether increasing Si concentrations (0, 1, 2, and 3 mmol L^−1^) supplied in the nutrient solution is uptaken by quinoa, modifies the C:N:P stoichiometry while increasing nutritional efficiency and crop productivity as well. Our results revealed that the Si supply by promoting a decline in the C levels, associated with greater uptake of N and P, especially decreased the C:N and C:P ratios, favoring the C metabolism efficiency, and modulated the N and P use efficiency for biomass accumulation. This improved nutritional performance and greater use efficiency of C directly favored quinoa productivity. The future perspective is to encourage new field studies with this species to adjust silicon fertilization management to different soils aiming at enhancing quinoa productivity on a sustainable basis.

## Introduction

Silicon (Si) as SiO_2_^1^ is relatively abundant (28.8%) in the earth's crust, but in plant dry mass is found predominantly as SiO_2_nH_2_O^2^, ranging from 1 to 5%. Furthermore, Si availability is relatively low in tropical soils. Generally, quinoa is expected to absorb Si since several plant species, such as eudicots and monocotyledons^3^, have been classified as intermediate and high Si accumulators, respectively. However, it remains unknown whether quinoa is a Si accumulator and/or beneficial to the species.

Si is considered an almost essential beneficial element for many plants due to its role in nutrient cycling within ecosystems^4^, modifying nutrient uptake, use efficiency, and nitrogen (N) and phosphorus (P) stoichiometry, as well as carbon (C) content^5–8^. Furthermore, the C:N:P stoichiometry is the most investigated factor in ecological interactions due to the strong link with the biochemical and physiological functioning of plants^9,10^.

Compared to C, Si incorporation by the plant tissue has a lower energy cost, probably indicating that Si could partially replace C in some plant organic compounds^11,12^. Previous studies suggest that increasing Si concentrations decreased C concentrations in rice^13^ and grasslands^8^, was also involved in the C and P metabolism in wheat plants, changed nutrient stoichiometry^5^, and the N and P^7^ metabolism in *Phragmintes australis*.

The Si impact on elemental stoichiometric ratios in different species may be related to the distinct uptake pattern of the nutrients since they depend on genes that encode transporters defined by genetic factors. Besides, the used Si source consists predominantly of pyrogenic silicon dioxide^5,7^, which despite the small particle size is non-soluble, unlike soluble sources such as potassium and sodium silicate that form monosilicic acid in solution and is readily absorbed by plants^14^. A study with sugarcane reported a greater Si uptake for the soluble source compared to silicon dioxide (nanosilica)^15^. Thus, further research using a soluble Si source should be conducted as it can improve plant Si uptake, its physiological responses, and elemental stoichiometry.

Si changes C metabolic efficiency and may improve the production of C skeletons in plants, affecting the metabolism and use efficiency of nutrients, such as N and P. In this sense, as N and P are also part of organic compounds that make up plants and various enzymes^16^, such as carriers, act as an energy source, consequently, altering the nutrient uptake and accumulation. Besides, N is intrinsically related to crop yield^17^ and plays an important role in the photosynthetic process^18^ and nutrient concentrations^19^. Although the Si and P interaction in physiological processes is doubtful, research indicates that P availability increased due to the Si effect on the expression of transporters and exudation of organic acids for P mobilization in the roots^20^. Thus, P and N together can influence productivity and biomass production^21^.

Quinoa is highlighted as the most complete vegetable because it contains all amino acids and has higher protein levels than cereals, based on dry matter^22^. In recent years, the interest in the quinoa crop has been growing due to the recognized quality of its grains, and the stress tolerance^23^ resulting from the fact that this crop originated in world regions difficult for plant growth. Because quinoa defense mechanisms may be associated with Si, its biological effects on this species should be further investigated.

Furthermore, the possible beneficial effects of Si are little known in plants that have not been investigated regarding its use^24^ yet. We hypothesized that Si supplied to quinoa should modify the stoichiometric C:N:P ratios, improve the nutrient content and use efficiency, thus increasing biomass production and productivity.

Given the above, our study aimed to assess whether soluble Si supplied via root is uptaken by quinoa, changes the C:N:P ratio, and increases crop nutritional efficiency and productivity.

## Results

### Si accumulation

**Figure 1:**
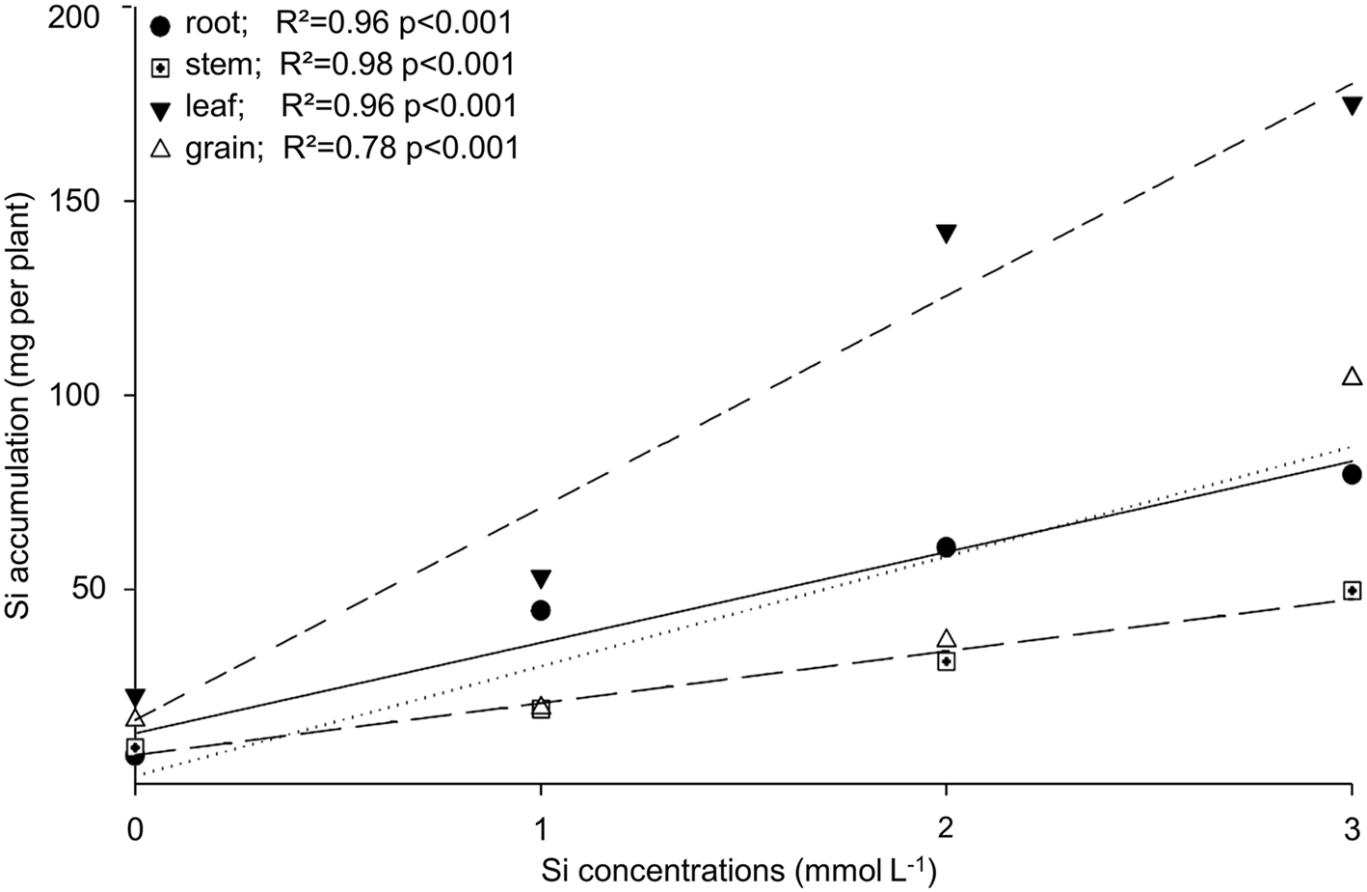
Si accumulation in the root, stem, leaf, and grains of quinoa for the studied Si concentrations.

As the supplied Si concentrations increased, Si accumulation increased linearly, based on the dry mass of different plant organs. For the 3 mmol L^-1^ Si concentration, Si accumulation per plant reached 175, 104, 79, and 49 mg in the leaf, grains, root, and stem, respectively. Therefore, at the highest Si concentration, the Si accumulation in the organs decreased as follows leaf > grain > root > stem (Fig. 1)**.**

### Concentrations of silicon and nutrients

The studied Si concentrations of 0, 1, 2, and 3 mmol L^−1^ are referred to as Si-0, Si-1, Si-2, and Si-3, respectively. Si supplied in the nutrient solution increased significantly (*p* < 0.05) the Si concentration in the evaluated plant organs (Supplementary Table S1).

**Figure 2:**
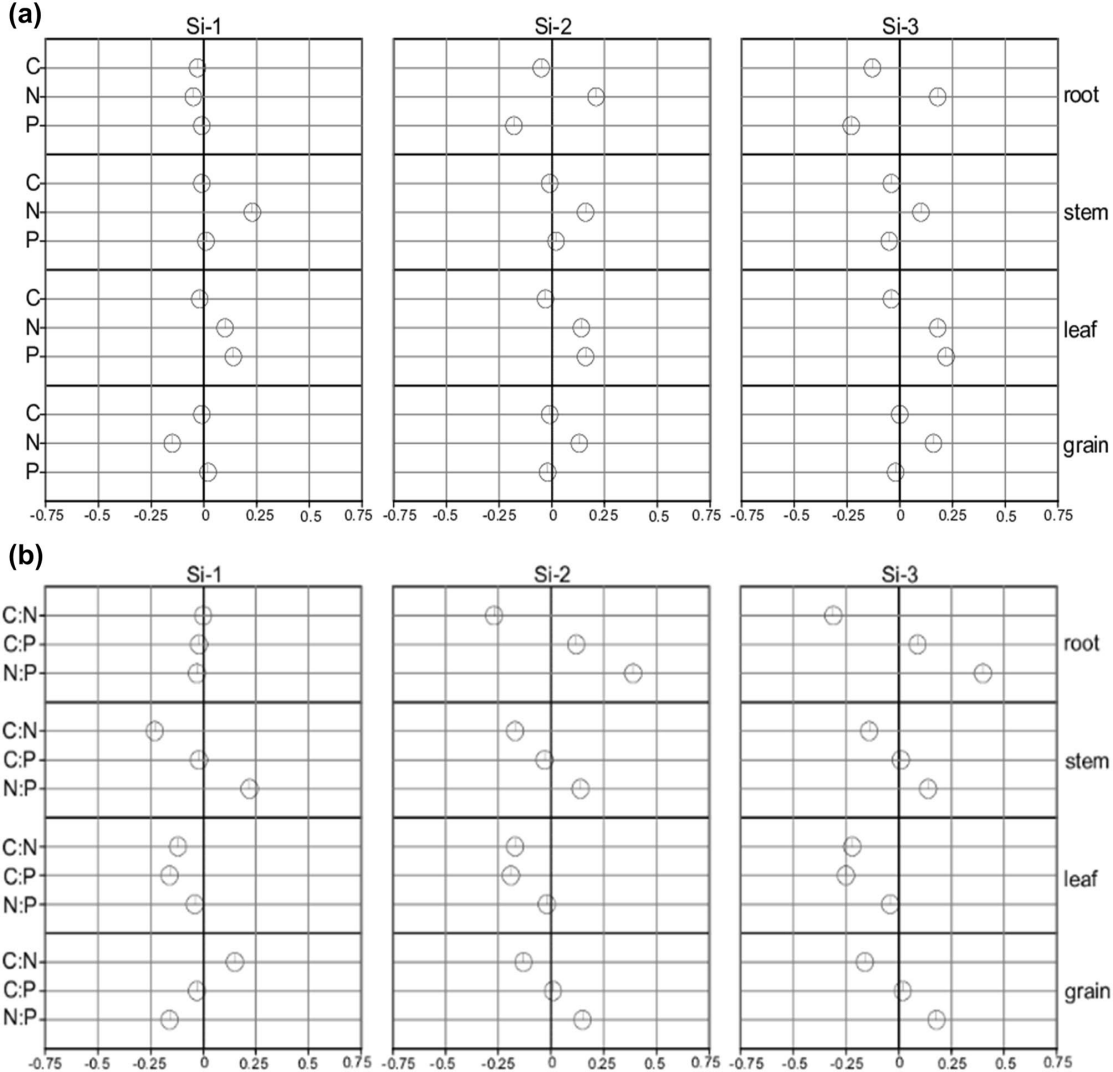
Changes [ln (x-Si-L/x-Si-0) ± ln (SD-Si-L/SD-Si-0), where x is the variable mean value and L is the Si addition level; n = 5] in carbon, nitrogen and phosphorus levels (**a**) and; C:N, C:P and N:P stoichiometric ratios (**b**) of analyzed organs for the studied Si concentrations.

Also, the supplied Si changed the carbon (C), nitrogen (N), and phosphorus (P) levels distinctly in the several plant organs. As the supplied Si increased, especially Si-3, the C content decreased by 11, 4 and 5%, in the root, stem and leaf, respectively (Fig. 2a, Supplementary Table S1).

**Figure 3:**
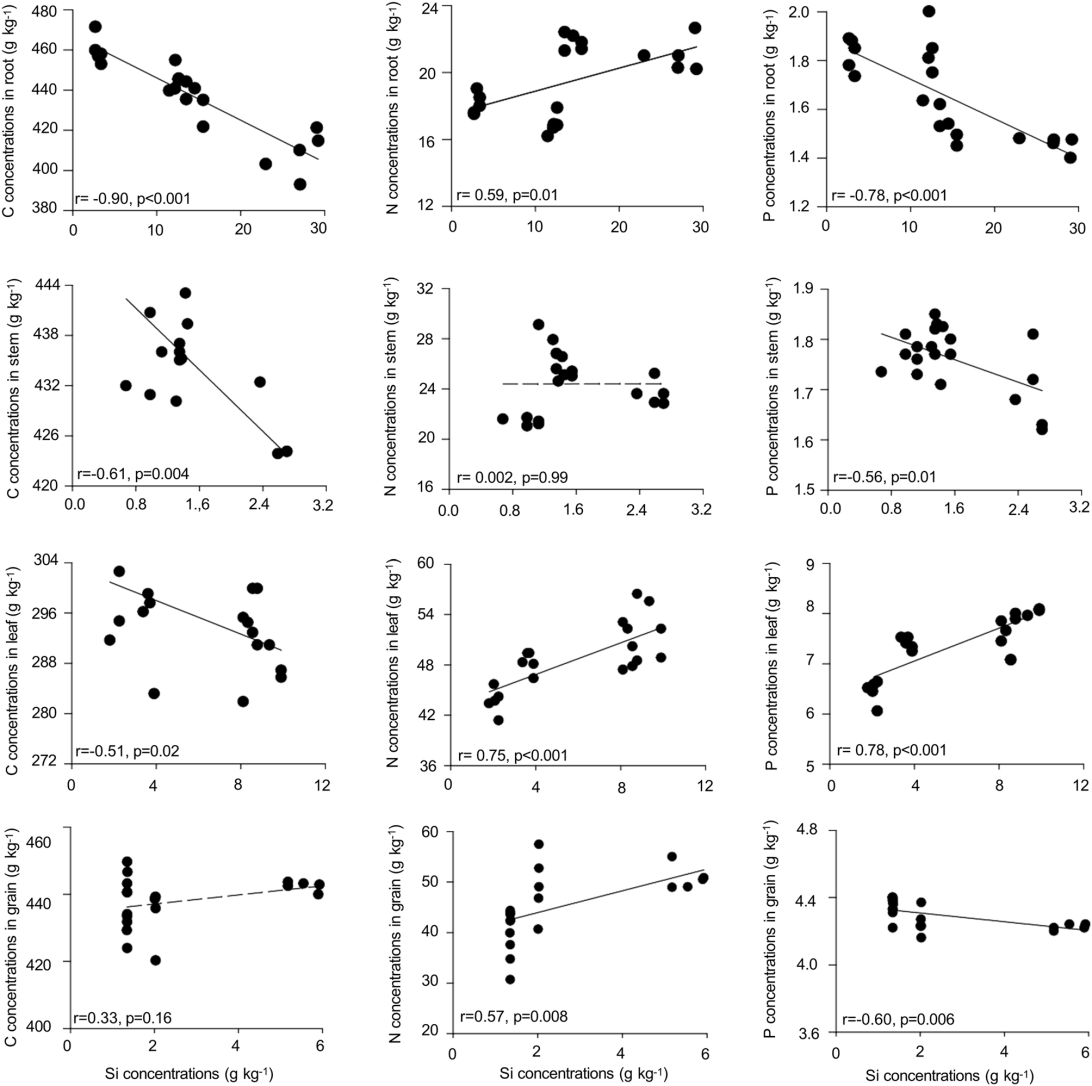
Correlation between Si concentrations in the root, stem, leaf, and grains of quinoa and nutrient concentrations (C, N, and P). Pearson's correlation coefficient (r) and the *p*-value are shown; significant correlations (*p* < 0.05) are indicated as black solid lines and non-significant correlations (*p* > 0.05) as dashed lines.

The results indicated that for all the plant organs, except for the stem, the concentrations of Si-2 and Si-3 promoted a 13 and 15% increase of N levels, respectively. The applied Si increased P content up to 19% in leaf tissue, while P availability decreased in the root and stem for Si-3, with the highest levels recorded for Si-0 (Fig. 3, Supplementary Table S1).

### C:N:P stoichiometric responses

Si application significantly altered all stoichiometric ratios (C:N, C:P and N:P), distinctly for each plant organ (Fig. 2b, Supplementary Table S1). Particularly for Si-2 and Si-3 concentrations, the root tissue exhibited a higher C:P (13 and 11%, respectively) and lower C:N (21 and 23%, respectively) stoichiometric ratios compared to the control (Si-0).

The C:N and N:P ratios in the stem decreased and increased by 12 and 16%, respectively. Further, in the leaves, the C:N ratio varied slightly (from 7 to 6) while the C:P ratio had a sharper decrease (from 47 to 36) as the applied Si concentrations increased. In the grains, the C:N ratio decreased by 14% between the highest Si concentration (Si-3) applied and the control (Si-0). The average values of the C:N and C:P ratios determined per plant organ decreased as follows root > stem > grains > leaf.

### Nutrient use efficiency

**Figure 4:**
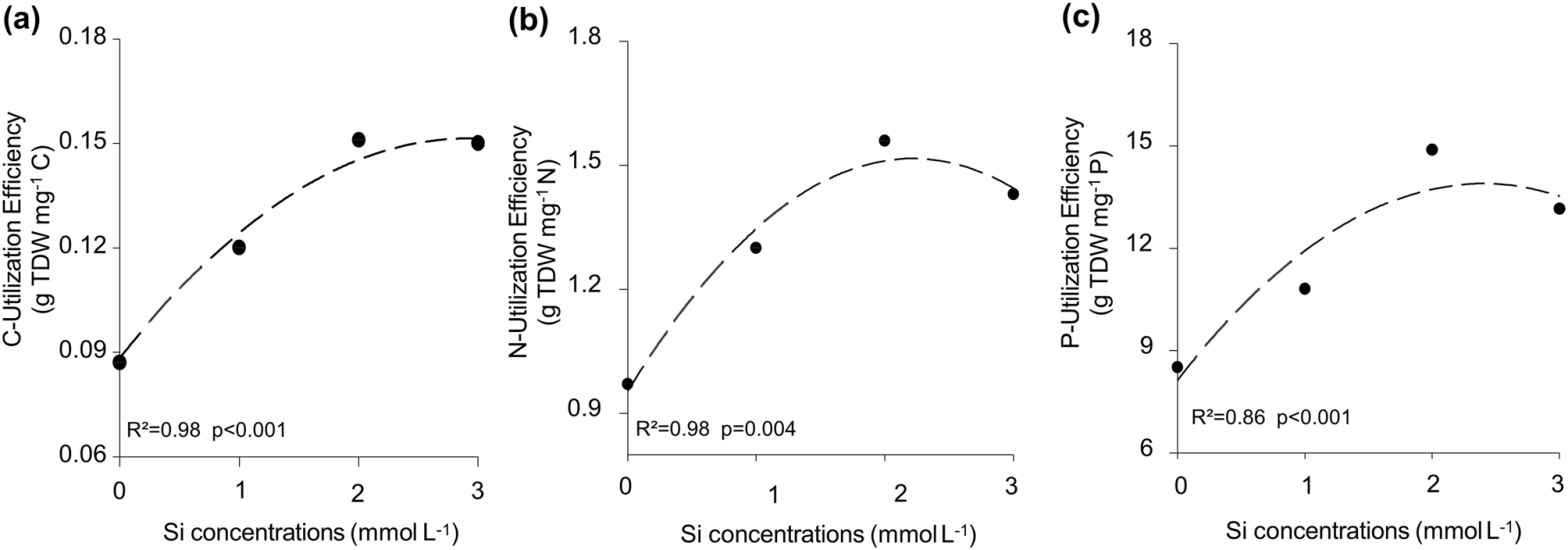
Use efficiency of carbon (**a**), nitrogen (**b**) and phosphorus (**c**) in quinoa plants for the studied Si concentrations.

Figure 4 shows that the C, N and P use efficiency increased following a quadratic fitting (Supplementary Table S2), for the supplied Si concentrations.

### Biomass production

**Figure 5:**
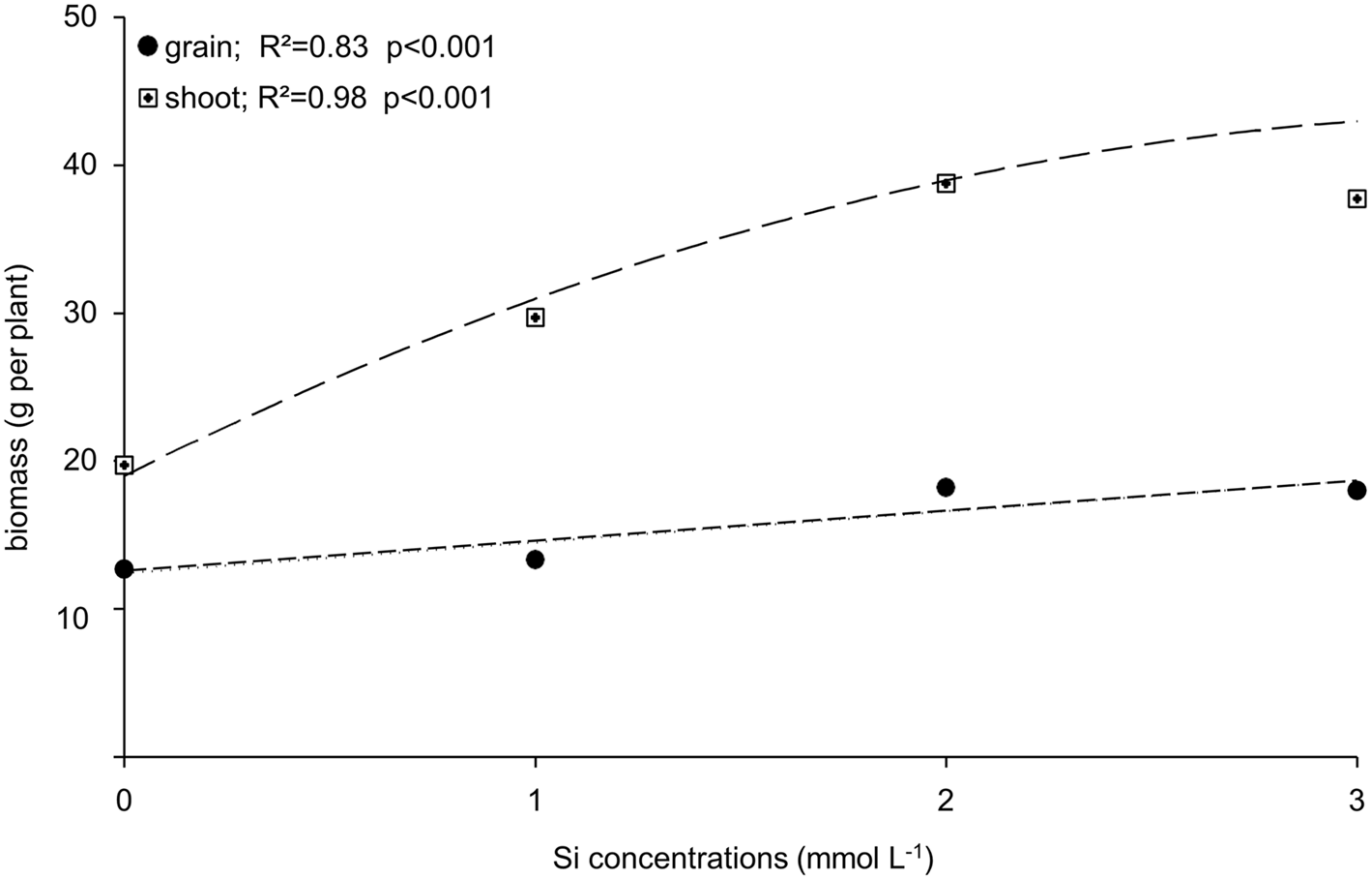
Shoot and grain biomass of quinoa for the studied Si concentrations.

The applied Si affected positively the biomass production of the shoot (stem and leaves), following a quadratic fitting and, consequently, a linear increase in the biomass of quinoa grains (Fig. 5, Supplementary Table S2). The Si application increased the grain biomass by approximately 45%.

## Discussion

The Si supplied in the nutrient solution via root increased Si accumulation in quinoa leaves (Fig. 1). Likewise, there are reports in the literature on the increasing Si levels in the leaves of crops such as wheat^5^, tomato^25^, and sugar cane^26^ for Si applied via root. The quinoa plants were able to transfer close to 43% of the total Si absorbed to the leaves, while the root and stem concentration was kept at relatively low levels. This higher Si level in the leaves compared to the root indicates that quinoa should be considered a Si-accumulating plant since it was able to accumulate more Si in the leaves than in the roots.

The results indicate that Si content in the quinoa leaves varied from 2.1 to 9.4 g kg^−1^ (Supplementary Table S1) depending on the applied Si concentration, this accumulation below 10 g kg^−1^ classifies quinoa as an intermediate Si accumulator^27^. Previous studies have shown that the Si uptake, as a monosilicic acid, by the roots of eudicots plants occurs passively, transported in the transpiratory flow, and permanently deposited as hydrated amorphous silica in the leaf tissue^3^. Thus, the supplied Si accumulated especially in the quinoa leaf tissue due to the intensity of the transpiration rate in the leaves and the balance between the Si available in the nutrient solution and the element demand by the plant^28^. The varying Si accumulation in quinoa grains may also depend on the level of Si available in the nutrient solution. Additionally, it has been suggested that the varying Si contents in grains can be genetically controlled^5,29^. We believe that this unprecedented result for quinoa should promote further research on the species and its interaction with Si.

The increased Si availability in quinoa organs alters the C, N and P contents, similar behavior has been reported for other plant species in studies that proved that Si partially controls the C flow while altering the N and P levels^8,30,31^. Our results indicate that increased Si supply reduced C content in most plant organs (Fig. 3), thus indicating a negative correlation between Si and C concentrations. The low C content in the tissues may indicate that the Si incorporated in the vegetal tissues replaced part of the C in the organic compounds of the cell wall, such as lignin and cellulose^7,32^. The energy cost for incorporating Si in structural compounds is less than the inclusion of C in organic compounds, due to the intrinsic permeability of the lipid bilayers^33^, also contributing to better plant growth. The decreasing C levels in different plant organs have not only been confirmed under normal growing conditions but also in plants grown under abiotic stress conditions, such as salinity^12,34^ and water deficit^35^, which induces greater energy expenditure by the plant and Si can mitigate this consumption, benefiting these plants.

The partial control of the C flow by Si modified the N and P concentrations, therefore, more N was available in the plant organs, except for the stem (Fig. 3). Our results demonstrate that the N content in the plant organs is affected by the supplied Si concentration, given the large difference observed between the moderate (Si-2) and high (Si-3) concentrations (Fig. 2a, Supplementary Table S1). In this sense, a greater Si availability can improve certain photosynthetic parameters and promote N uptake, as indicated in previous research^26^, contradicting other results that reported a negative correlation between N and Si in the leaf tissue^36^. Also, it is possible to suggest the possible role of Si in reducing the permeability of the plasma membrane, increasing root activity, and resulting in higher nutrient uptake^33^.

Furthermore, P concentrations increased only in the leaves with added Si (Fig. 3), suggesting a possible nutrient translocation from the root and stem to this tissue. A similar result was also reported for wheat^5^ and sugar cane^35^, where adding Si in the nutrient solution increased P availability in the leaves while Si involvement in the metabolism of C and P was indicated, with subsequent changes in the nutrient stoichiometry and use efficiency. Studies in the literature suggest that Si changes the positive regulation of the expression of transporter genes for P uptake while increasing the exudation rates of organic acids that play a role in improving P uptake and availability^20^.

Changes in nutrient concentrations interfered with the nutrient ratios in the plant. Stoichiometric ratios of nutrients are the object of study because the nutrient concentration in plant tissues is affected by biomass production; also interfering in ecological interactions due to the important role of these nutrients in the biological and biochemical functions of plants^37^. Our results indicated that Si replaces and decreases C, increases the N availability, while the P concentrations differ for each plant organ. This result indicates that the C:N and C:P ratios changed in the different organs, while the N:P ratio was more representative of the grains (Fig. 2b). The C:N ratio in the different tissues varied because the applied Si caused the C levels to decrease followed by increasing N contents. However, the decrease of the C:N ratio in the leaves was more pronounced because the C concentration in this organ decreased more significantly, while no significant trend was observed in the grain C contents. The general decreasing pattern of the C:N ratios due to increasing Si concentrations may be related to the decreasing C levels in structural compounds, as previously indicated^7,8^.

The C:N and C:P ratios represent the plant capacity for photosynthetic fixation of C through the availability of N or P^38^. Therefore, the higher N content in the leaf tissue demonstrated its role in the photosynthetic activity of fixing the C that forms part of the plant organic compounds^18^. The supplied Si decreased C while increasing P content, thus decreasing the C:P ratio in the leaves (Fig. 2b). However, the N:P ratio changed especially due to the greater availability of N in the leaf and grains, interfering with plant biomass production and composition, as previously indicated^9^. The lower C:N and C:P ratios point to the role Si plays in maintaining the balance between C and nutrients, N and P^39^. To this end, the increased Si uptake and accumulation contributes to maintain and regulate the homeostasis of stoichiometric compositions in tissues, increasing the structural compounds of C with important metabolic function by favoring the physiological and biochemical processes of the plant.

Our research indicates that a better understanding of the Si effects on quinoa growth and stoichiometric C:N:P ratios requires considering the different responses to the nutrient use efficiency (Fig. 4). The use efficiency of C increased from 0.087 ± 0.003 (Si-0) to 0.120 ± 0.003 (Si-1), 0.154 ± 0.002 (Si-2) and decreased to 0.151 ± 0.00 (Si-3). This result is attributed to higher Si incorporation by the plant tissues that acted mainly in the photosynthetic process instead of replacing the C of the organic compounds^7,35^. The supplied Si also affected N use efficiency by increasing biomass production when N concentration in the plant organs was higher as well. The N use efficiency increased from 0.97 ± 0.03 (Si-0) to 1.30 ± 0.05 (Si-1), 1.56 ± 0.05 (Si-2), and decreased to 1.43 ± 0.02 (Si-3). This increased N use efficiency probably resulted from changes in the primary metabolism, which stimulated the translocation of amino acids to the absorbing tissues^40^, supported also by the greater equilibrium in the composition of amino acids in the quinoa tissues^41^. Similar results have been previously reported for wheat plants^5^, indicating a significant increase in biomass production. The P use efficiency increased from 8.50 ± 0.24 (Si-0) to 10.80 ± 0.33 (Si-1), 14.88 ± 0.29 (Si-2), and decreased to 13.15 ± 0.09 (Si-3). This referred increase may have been due to the reduced energy cost in the substitution of Si for C in organic compounds.

Hence, the results show that Si improved the C, N, and P use efficiency since the plant produced more biomass per unit of N and P absorbed and reduced the C:N and C:P ratios, allowing us to accept our hypothesis that Si increases the use efficiency of C, N and P. Si promoted high nutritional efficiency with contributions from physiological processes because it is known that Si increases photosynthetic efficiency and water use^42^. Therefore, the greater N availability caused biomass production to increase in plant organs related to growth and storage such as leaves and grains since, in these tissues, C compounds were partially replaced by Si, in addition to P translocation and accumulation; these results are supported by previous research in other species^5,7,26^. Thus, the Si absorbed by the quinoa plants changed the C:N and C:P stoichiometric ratios, especially, by increasing the use efficiency of C, N, and P with a subsequent increase of biomass and productivity observed for the added 2.7 and 3 mmol L^−1^ Si concentrations, respectively.

In conclusion, these results demonstrate that quinoa Si uptake and accumulation decreases as follows leaf > grain > root > stem, whereas Si availability in the plant increases the biomass and grain productivity by changing the nutrient stoichiometric ratios, i.e., decreasing C concentrations and improving P and N use efficiency. Therefore, highlighting the biological importance of Si to quinoa plants, which are very tolerant of different stresses and present a high nutritional value, we can infer that possibly the plant defense mechanisms are strongly associated with Si, thus, indicating the need for further studies with this species on different topics related to plant nutrition.

## Methods

### Experimental conditions

The experiment was conducted in a hydroponic growing system in a greenhouse at the São Paulo State University, in Jaboticabal, Brazil. Seeds of quinoa cv. BRS Piabiru^43^, were obtained from the Brazilian Agricultural Research Corporation of the Ministry of Agriculture, Livestock and Food Supply, Brazil. This research was not conducted with endangered species and was conducted in accordance with the is in accordance with the Declaration of IUCN Policy on Research Involving Endangered Species. During the experiment, the maximum and minimum temperatures and relative humidity were measured daily using a thermohygrometer (Supplementary Figure S1). The experiment was carried out as randomized blocks, with four treatments and five repetitions. The Si concentrations of 0 (Si-0), 1 (Si-1), 2 (Si-2), and 3 (Si-3) mmol L^-1^ were supplied in the nutritive solution via root, using potassium silicate and sodium stabilized with sorbitol as Si sources [Si = 107.9 g L^−1^; K_2_O = 16.44 g L^−1^; Na_2_O = 60.7 g L^−1^]. The K concentration in the nutrient solution was adjusted with potassium chloride in all treatments.

Ten quinoa seeds were sown in plastic pots containing 6 dm^3^ of medium-grained sand, which was previously washed with running water, and then with deionized and distilled water^44^. As plants developed, only one plant per pot was maintained. After emergence, Si was added to the modified nutrient solution^45^, Fe source changed from Fe-EDTA to Fe-EDDHMA. The nutrient solution pH was adjusted to 5.5 ± 0.2, using a 1.0 mol L^−1^ solution of either HCl or NaOH. Sorbitol plays an indispensable role as Si stabilizer in aqueous solutions by decreasing the polymerization process at pH below 7. The ionic concentration of the nutrient solution was increased during the culture cycle, starting with 10% during the first 10 days after emergence, followed by 25% in leaf development (up to six true leaves), 50% since the emergence of inflorescence, 80% in flowering and 100% in fruit development until the end of the experiment^46^.

### Plant analysis

When the plants reached physiological maturity, the biomass was separated into root, stem, leaves, and grains. All plant tissues were washed in deionized water with a detergent solution (0.1%), HCl solution (0.3%), and deionized water. The plant material was dried in a forced-air circulation oven at 65 ± 5 °C to constant weight, and dry weight was determined.

The C and N contents in the dry mass were determined by the dry combustion method (1000 °C) in an elemental analyzer (LECO Truspec CHNS) calibrated with the LECO 502-278 wheat standard (C = 45.00% and N = 2.68%). Total P concentrations were measured using the molybdenum antimony colorimetric method^47^. The Si content was determined by wet digestion, hydrogen peroxide (H_2_O_2_) and sodium hydroxide (NaOH) were added, and the reaction was induced in an autoclave at 123 °C. The silicon was determined by the colorimetry method^48^ with hydrochloric acid, oxalic acid and ammonium molybdate in a spectrophotometer at 410 nm.

From the nutrient concentrations in the plant tissue (g kg^−1^) and dry matter (g per plant), the nutrient accumulation was calculated and expressed as mg per plant. The C:N:P stoichiometric ratios were determined in different plant organs and expressed in g kg^-1^. Nutrient use efficiency was calculated as the total plant dry weight (TDW) divided by the C, N and P contents (g TDW mg^−1^ of each element)^49^:

### Statistical analysis

After testing normality (Kolmogorov–Smirnov test) and homogeneity of variance (Shapiro–Wilk test), all data were submitted to analysis of variance and, when the F test was significant (*p* < 0.05), the data were fitted to the linear or quadratic polynomial regression model opting for the model with the highest coefficient of determination (R^2^). Pearson's correlation test was performed for the sample size n = 20. The statistical analyses were performed using SAS Version 9.1 (SAS Institute, Cary, NC, USA). The log response values for the numbers were calculated by ln of the mean values (Si treatment/control) in the following equation^50^: ln [${\overline{\text{X}}}$_(Si-treatment)_/${\overline{\text{X}}}$_(control)_) ± (s^2^_(Si-treatment)_/n _(Si-treatment)_ ${\overline{\text{X}}}^{2}$_(Si-treatment)_ + (s^2^_(control)_/n _(control)_ ${\overline{\text{X}}}^{2}$_(control)_]. If the ln response rate is 0, the Si treatments and control do not differ. If it is less than 0, the Si treatments are lower than the control and if the ln response rate is greater than zero, the Si treatments are higher than the control.

## Supplementary Information

Supplementary Information.

## References

[1] Hans WK (1995). The composition of the continental crust. Geochim. Cosmochim. Acta..

[2] Birchall JD (1995). The essentiality of silicon in biology. Chem. Soc. Rev..

[3] Kaur H, Greger M (2019). A review on si uptake and transport system. Plants.

[4] Struyf E (2011). Tracing Si-N-P ecosystem-pathways: Is relative uptake in riparian vegetation influenced by soil waterlogging, mowing management and species diversity?. Hydrobiologia.

[5] Neu S, Schaller J, Dudel EG (2017). Silicon availability modifies nutrient use efficiency and content, C:N: P stoichiometry, and productivity of winter wheat (*Triticum aestivum* L).. Sci. Rep..

[6] Pontigo S (2015). Silicon in vascular plants: Uptake, transport and its influence on mineral stress under acidic conditions. Planta.

[7] Schaller J, Brackhage C, Gessner MO, Bäuker E, Gert Dudel E (2012). Silicon supply modifies C:N: P stoichiometry and growth of *Phragmites australis*. Plant Biol..

[8] Hao Q (2020). Silicon affects plant stoichiometry and accumulation of C, N, and P in grasslands. Front. Plant Sci..

[9] Güsewell S (2004). N: P ratios in terrestrial plants: variation and functional significance. New Phytol..

[10] Sardans J, Peñuelas J (2012). The role of plants in the effects of global change on nutrient availability and stoichiometry in the plant-soil system. Plant Physiol..

[11] Schaller J, Brackhage C, Dudel EG (2012). Silicon availability changes structural carbon ratio and phenol content of grasses. Environ. Exp. Bot..

[12] Liu L (2020). Silicon effects on biomass carbon and phytolith-occluded carbon in grasslands under high-salinity conditions. Front. Plant Sci..

[13] Klotzbücher T (2018). Variable silicon accumulation in plants affects terrestrial carbon cycling by controlling lignin synthesis. Glob. Chang. Biol..

[14] Kudryavtsev PG, Figovsky O (2016). Nanocomposite organomineral hybrid materials. Nanotechnol. Constr..

[15] Santos LCN, Teixeira GCM, Prado RM, Rocha AMS, Pinto RCS (2020). Response of pre-sprouted sugarcane seedlings to foliar spraying of potassium silicate, sodium and potassium silicate, nanosilica and monosilicic acid. Sugar Tech..

[16] Krouk G, Kiba T (2020). Nitrogen and phosphorus interactions in plants: From agronomic to physiological and molecular insights. Curr. Opin. Plant Biol..

[17] Basra SMA, Iqbal S, Afzal I (2014). Evaluating the response of nitrogen application on growth, development and yield of quinoa genotypes. Int. J. Agric. Biol..

[18] Sun J, Ye M, Peng S, Li Y (2016). Nitrogen can improve the rapid response of photosynthesis to changing irradiance in rice (*Oryza sativa* L.) plants. Sci. Rep..

[19] Zahoor (2014). Role of nitrogen fertilizer in crop productivity and environmental pollution. Int. J. Agric. For..

[20] Kostic L, Nikolic N, Bosnic D, Samardzic J, Nikolic M (2017). Silicon increases phosphorus (P) uptake by wheat under low P acid soil conditions. Plant Soil.

[21] Guignard MS (2017). Impacts of nitrogen and phosphorus: From genomes to natural ecosystems and agriculture. Front. Ecol. Evol..

[22] Silva PM (2020). Quinoa (*Chenopodium quinoa* Willd.): an overview of the potentials of the “golden grain” and socio-economic and environmental aspects of its cultivation and marketization. Foods.

[23] Hinojosa L, González JA, Barrios-Masias FH, Fuentes F, Murphy KM (2018). Quinoa abiotic stress responses: a review. Plants.

[24] Ma JF, Yamaji N (2006). Silicon uptake and accumulation in higher plants. Trends Plant Sci..

[25] Al-aghabary K, Zhu Z, Shi Q (2004). Influence of silicon supply on chlorophyll content, chlorophyll fluorescence, and antioxidative enzyme activities in tomato plants under salt stress. J. Plant Nutr..

[26] Frazão JJ, Prado RM, Souza JP, Rossatto DR (2020). Silicon changes C:N: P stoichiometry of sugarcane and its consequences for photosynthesis, biomass partitioning and plant growth. Sci. Rep..

[27] Takahashi E, Ma JF, Miyake Y (1990). The possibility of silicon as an essential element for higher plants. Comments Agric. Food Chem..

[28] Faisal S, Callis KL, Slot M, Kitajima K (2012). Transpiration-dependent passive silica accumulation in cucumber (*Cucumis sativus*) under varying soil silicon availability. Botany.

[29] Ma JF, Higashitani A, Sato K, Tateda K (2003). Genotypic variation in Si content of barley grain. Plant Soil.

[30] Greger M, Landberg T, Vaculík M (2018). Silicon influences soil availability and accumulation of mineral nutrients in various plant species. Plants.

[31] Long M (2018). Effects of water and exogenous Si on element concentrations and ecological stoichiometry of plantain (*Plantago lanceolata* L.). J. Plant Nutr..

[32] Schoelynck J (2010). Silica uptake in aquatic and wetland macrophytes: A strategic choice between silica, lignin and cellulose?. New Phytol..

[33] Raven J (1983). The transport and function of silicon in plants. Biol. Rev..

[34] Hurtado A (2020). Silicon application induces changes C:N:P stoichiometry and enhances stoichiometric homeostasis of sorghum and sunflower plants under salt stress. Saudi J. Biol. Sci..

[35] Teixeira GCM, Prado RM, Rocha AMS, Cássia Piccolo M (2020). Root- and foliar-applied silicon modifies C:N:P ratio and increases the nutritional efficiency of pre-sprouted sugarcane seedlings under water deficit. PLoS ONE.

[36] Murozuka E (2014). Nitrogen fertilization affects silicon concentration, cell wall composition and biofuel potential of wheat straw. Biomass Bioenerg..

[37] Prado, R. M. & Pereira, G. Ecological response to global change: Changes in C:N:P stoichiometry in environmental adaptations of plants. in *Plant Ecology: Traditional Approaches to Recent Trends* 2–19. 10.5772/intechopen.69246 (2017).

[38] Koerselman W, Meuleman AFM (2019). The vegetation N:P ratio: a new tool to detect the nature of nutrient limitation. J. Appl. Ecol..

[39] Viciedo DO, Prado RM, Martínez CA, Habermann E, Cássia Piccolo M (2019). Short-term warming and water stress affect *Panicum maximum* Jacq. stoichiometric homeostasis and biomass production. Sci. Total Environ..

[40] Detmann KC, Araújo WL, Martins SCV, Fernie AR, DaMatta FM (2013). Metabolic alterations triggered by silicon nutrition: Is there a signaling role for silicon?. Plant Signal. Behav..

[41] Thanapornpoonpong SN, Vearasilp S, Pawelzik E, Gorinstein S (2008). Influence of various nitrogen applications on protein and amino acid profiles of amaranth and quinoa. J. Agric. Food Chem..

[42] Meunier JD (2017). Effect of phytoliths for mitigating water stress in durum wheat. N. Phytol..

[43] Spehar CR (2006). Adaptation of quinoa (*Chenopodium quinoa* Willd.) to increase the agricultural and alimentary diversity in Brazil. Cad. Ciência Tecnol..

[44] Filho LO, Silva MS, Vareiro WP, Zanutto RP (2018). Limpeza de areia para experimentos em nutrição de plantas.

[45] Hoagland, D. & Arnon, D. The water-culture method for growing plants without soil. *Circ. Calif. Agric. Exp. Stn*. **347**, (1950).

[46] Sosa-Zuniga V, Brito V, Fuentes F, Steinfort U (2017). Phenological growth stages of quinoa (*Chenopodium quinoa*) based on the BBCH scale. Ann. Appl. Biol..

[47] Bataglia, O., Teixeira, J., Furlani, P. & Furlani, A. *Métodos de análise química de plantas* (1983).

[48] Korndörfer G, Pereira H, Nolla A (2004). Análise de silício: solo, planta e fertilizante.

[49] Siddiqi MY, Glass ADM (1981). Utilization index: a modified approach to the estimation and comparison of nutrient utilization efficiency in plants. J. Plant Nutr..

[50] Hedges LV, Gurevitch J, Curtis PS (1999). The meta-analysis of response ratios in experimental ecology. Ecology.

