# Supplementary material for: Silicon modifies C:N:P stoichiometry, and increases nutrient use efficiency and productivity of quinoa
Source: Sci Rep. 2021 May 10;11:9893. doi: 10.1038/s41598-021-89416-9 (PMC8110966; doi:10.1038/s41598-021-89416-9)
Supplement: Supplementary file 1 — Supplementary Information. [file 41598_2021_89416_MOESM1_ESM.pdf]

Silicon modifies C:N:P stoichiometry, and increases nutrient use efficiency and productivity of quinoa

Luis Felipe Lata-Tenesaca[1 *], Renato de Mello Prado[1], Marisa de Cássia Piccolo[2], Dalila Lopes da Silva[1], José Lucas Farias da Silva[1]

[1] São Paulo State University (UNESP), School of Agricultural and Veterinarian Sciences, Jaboticabal, São Paulo, 14884-900, Brazil.

[2] University of São Paulo, Center of Nuclear Energy in Agriculture, Piracicaba, São Paulo, 13400-970, Brazil

*Correspondence and requests for materials should be addressed to L.F. Lata-Tenesaca (email: luis_lt22@outlook.com).

Supplementary Figures

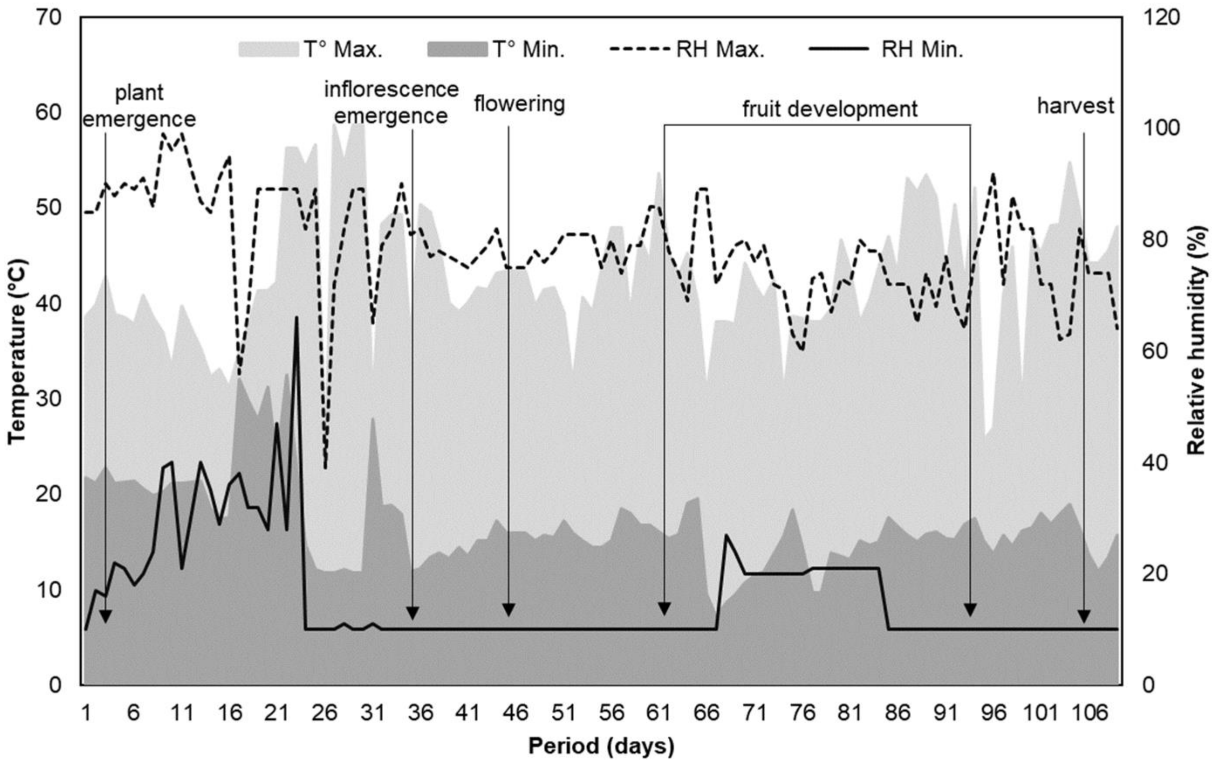

Figure S1. Experimental conditions in the greenhouse during the experiment. Maximum (T° Max.) and minimum (T° Min.) temperatures, and maximum (RH Max.) and minimum (RH Min.) relative humidity.

Supplementary Tables

Table S1. Varying Si and nutrient concentrations, and C:N:P stoichiometry in quinoa tissue (g kg^{-1}). Values are presented as means ± SE (n=5). Different letters mean significant differences between Si treatments (^{abcd} for p<0.001, ^{ef} for p<0.05) while same letters mean no significant differences between treatments (p>0.05).

		Root	Stem	Leaf	Grain
**Silicon**	Si-0	3.04 ± 0.27 c	0.98 ± 0.06 c	2.08 ± 0.1 d	1.35 ± 0.04 c
	Si-1	12.22 ± 0.33 b	1.31 ± 0.02 b	3.70 ± 0.1 c	1.35 ± 0.04 c
	Si-2	14.53 ± 0.58 b	1.46 ± 0.04 b	8.33 ± 0.13 b	2.02 ± 0.04 b
	Si-3	27.07 ± 0.92 a	2.59 ± 0.07 a	9.35 ± 0.23 a	5.55 ± 0.12 a
**Carbon**	Si-0	459.83 ± 2.49 a	440.74 ± 2.59 e	302.03 ± 4.32a	440.48 ± 2.34 a
	Si-1	445.23 ± 3.84 ab	436.06 ± 2.01 e	296.22 ± 3.80ab	434.01 ± 3.26 a
	Si-2	435.43 ± 3.19 b	435.22 ± 2.04 e	292.90 ± 2.22ab	434.53 ± 3.31 a
	Si-3	408.40 ± 3.07 c	424.09 ± 1.25 f	286.09 ± 1.57 b	442.37 ± 1.35 a
**Nitrogen**	Si-0	18.13 ± 0.64 b	23.98 ± 0.37 a	43.68 ± 0.48 f	43.24 ± 0.17 ef
	Si-1	16.91 ± 0.31 b	25.65 ± 0.53 a	48.32 ± 0.88 ef	37.34 ± 2.23 f
	Si-2	21.82 ± 0.2 a	25.14 ± 0.14 a	50.16 ± 1.13 e	49.29 ± 2.69 e
	Si-3	21.03 ± 0.47 a	24.63 ± 0.27 a	52.33 ± 1.3 e	50.83 ± 1.09 e
**Phosphorus**	Si-0	1.83 ± 0.02 a	1.77 ± 0.01 ef	6.45 ± 0.10 c	4.31 ± 0.02 ab
	Si-1	1.81 ± 0.04 a	1.79 ± 0.02 ef	7.41 ± 0.08 b	4.39 ± 0.01 a
	Si-2	1.53 ± 0.04 b	1.80 ± 0.02 e	7.42 ± 0.13 b	4.25 ± 0.03 bc
	Si-3	1.46 ± 0.02 b	1.69 ± 0.03 f	8.0 ± 0.01 a	4.22 ± 0.01 c
**C:N**	Si-0	25.39 ± 0.50 a	20.61 ± 0.12 a	6.92 ± 0.04 a	10.19 ± 0.06 ef
	Si-1	26.35 ± 0.36 a	15.92 ± 0.26 c	6.13 ± 0.1 b	11.79 ± 0.61 e
	Si-2	19.96 ± 0.20 b	17.32 ± 0.12 b	5.85 ± 0.15 b	8.95 ± 0.6 f
	Si-3	19.45 ± 0.34 b	17.97 ± 0.33 b	5.58 ± 0.16 b	8.72 ± 0.18 f
**C:P**	Si-0	252.0 ± 2.71 b	249.67 ± 2.82 a	46.88 ± 0.94 a	102.21 ± 0.79 ab
	Si-1	247.03 ± 3.60 b	244.49 ± 4.05 a	39.99 ± 0.93 b	99.19 ± 0.82 b
	Si-2	285.49 ± 6.89 a	241.94 ± 1.81 a	39.55 ± 0.90 b	102.22 ± 0.94 ab
	Si-3	280.32 ± 4.07 a	251.12 ± 4.09 a	36.36 ± 0.26 b	104.73 ± 0.43 a
**N:P**	Si-0	9.93 ± 0.17 b	12.11 ± 0.10 c	6.78 ± 0.11 a	10.03 ± 0.06 ef
	Si-1	9.38 ± 0.19 b	15.38 ± 0.27 a	6.52 ± 0.14 a	8.51 ± 0.50 f
	Si-2	14.31 ± 0.39 a	13.97 ± 0.14 b	6.76 ± 0.14 a	11.59 ± 0.62 e
	Si-3	14.44 ± 0.40 a	13.99 ± 0.32 b	6.54 ± 0.15 a	12.03 ± 0.26 e

Table S2. Si accumulation equations parameters; C, N, and P concentrations and use efficiency; and biomass of quinoa organs for the studied Si concentrations.

Organs[(1)]	Parameters	Si Accumulation	Use efficiency nutrient			Biomass
			C	N	P	
Root	Coefficient (a)<br>Coefficient (b)<br>R^2	23.33<br>12.98<br>0.96	-	-	-	-
Stem	Coefficient (a)<br>Coefficient (b)<br>R^2	13.36<br>7.39<br>0.98	-	-	-	-
Leaf	Coefficient (a)<br>Coefficient (b)<br>R^2	54.62<br>16.42<br>0.96	-	-	-	-
Grain[(2)]	Coefficient (a)<br>Coefficient (b)<br>R^2	28.25<br>1.96<br>0.78	-	-	-	2.09<br>14.41<br>0.83
Whole plant	Coefficient (a)<br>Coefficient (b)<br>Constant (c)<br>R^2	-	-0.009<br>0.07<br>0.03<br>0.98	-0.11<br>0.74<br>0.34<br>0.97	-1.01<br>4.83<br>8.12<br>0.86	-
Shoot[(3)]	Coefficient (a)<br>Coefficient (b)<br>Constant (c)<br>R^2	-	-	-	-	-2.75<br>14.57<br>19.27<br>0.98

[(1)] Means of analyzed organs; [(2)] Equation: $y= ax+b$.
[(3)] Equation: $y = ax^2+bx+c$, model not fitted (-).
